# Presenting symptoms and diagnostic accuracy of prehospital stroke scales for patients with suspected mild minor stroke

**DOI:** 10.1093/esj/23969873251360592

**Published:** 2026-01-01

**Authors:** Helge Fagerheim Bugge, Mona Guterud, Karianne Larsen, Mathias Toft, Maren Ranhoff Hov, Else Charlotte Sandset

**Affiliations:** Institute of Clinical Medicine, Faculty of Medicine, University of Oslo, Oslo, Norway; Department of Research, Norwegian Air Ambulance Foundation, Oslo, Norway; Department of Neurology, Oslo University Hospital, Oslo, Norway; Department of Research, Norwegian Air Ambulance Foundation, Oslo, Norway; Department of Research, Norwegian Air Ambulance Foundation, Oslo, Norway; Department of Neurology, Oslo University Hospital, Oslo, Norway; Institute of Clinical Medicine, Faculty of Medicine, University of Oslo, Oslo, Norway; Department of Neurology, Oslo University Hospital, Oslo, Norway; Department of Research, Norwegian Air Ambulance Foundation, Oslo, Norway; Department of Neurology, Oslo University Hospital, Oslo, Norway; Department of Health Science, Oslo Metropolitan University, Oslo, Norway; Institute of Clinical Medicine, Faculty of Medicine, University of Oslo, Oslo, Norway; Department of Research, Norwegian Air Ambulance Foundation, Oslo, Norway; Department of Neurology, Oslo University Hospital, Oslo, Norway

**Keywords:** Minor stroke, prehospital, symptoms, NIHSS

## Abstract

**Introduction:**

Identifying patients with minor stroke is challenging in the prehospital setting due to subtle symptoms. The majority of studies evaluating prehospital stroke scales include patients with high median NIHSS at admission. ParaNASPP, a stepped-wedge cluster-randomized controlled trial found that prehospital NIHSS identified more patients with minor symptoms. Further knowledge on presenting symptoms of patients with suspected minor stroke, and the accuracy of prehospital stroke scales on minor stroke is needed.

**Methods:**

A post-hoc analysis of data from the ParaNASPP trial describes prehospital presenting signs and symptoms of patients with suspected mild minor stroke. We defined mild minor stroke as NIHSS 0–2 at hospital admission. Furthermore, we reconstructed and evaluated nine prehospital stroke scales (NIHSS, FAST/CPSS, BE-FAST, LAPSS, MASS, MedPacs, PreHAST, and sNIHSS-EMS) in patients with mild minor stroke.

**Results:**

Four hundred and thirty-one patients in the ParaNASPP trial had NIHSS 0–2 at hospital admission. Of these, 152 (35%) were discharged from hospital with a stroke diagnosis. When examined by paramedics, stroke patients presented with speech disturbance, facial palsy, and motor weakness in arm or leg, while stroke mimics presented with dizziness, headache, and nausea/vomiting. NIHSS had the highest sensitivity (95%) and lowest specificity (16%), while LAPSS had the lowest sensitivity (42%) and highest specificity (80%) in the patients with suspected mild minor stroke. The remaining scales had sensitivity between 67% and 93%, and specificity between 23% and 67%.

**Conclusions:**

In patients with mild minor stroke, substantial overlap in presentation between stroke and stroke mimics makes triage challenging. Prehospital stroke scales provide either high sensitivity or specificity. Competence and training of paramedics in when and how to use, and interpret, these scales is key for recognizing and correctly triaging stroke patients.

The ParaNASPP trial was registered at Clinicaltrials.gov with registration number NCT04137874.

## Graphical Abstract

**Graphical abstract:**
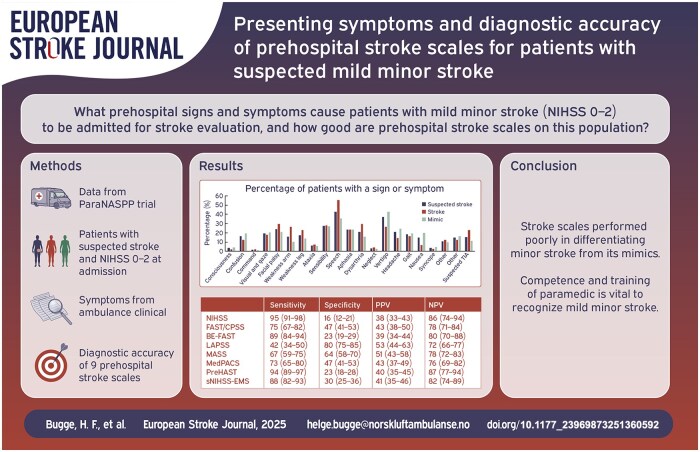

## Introduction

Nearly half of all stroke cases worldwide are classified as minor stroke, and as many as two-thirds of patients suffering an ischemic stroke present with mild symptoms.^1–3^ Although the prognosis of patients suffering a minor stroke is better than for patients with more severe strokes, minor stroke may still lead to significant disability and even death.^4^ Several studies have shown that dual antiplatelet treatment (DAPT) is a treatment option for non-disabling minor stroke of non-cardioembolic etiology.^5–8^ However, the role of reperfusion treatment in patients with minor stroke is uncertain. Current guidelines recommend reperfusion treatment with intravenous thrombolysis for patients suffering a disabling minor stroke.^9^ The definition of a disabling stroke is much left to the treating stroke physician and will vary according to individual patients. Therefore, timely identification of patients presenting with symptoms of minor stroke is key to decide and initiate correct treatment and secondary prevention.

Prehospital identification and correct triage of patients with stroke is challenging, and suffering a mild stroke is a predictor of prehospital delay.^10^ Since stroke has a wide range of presenting symptoms, with limited diagnostic possibilities in the prehospital setting, paramedics rely on stroke scales with suboptimal diagnostic accuracy.^11,12^ Prehospital stroke scales are designed for rapid and simple use, aiming for identification of the most common stroke symptoms.^12,13^

Most research on prehospital stroke scales has been performed in populations with moderate to severe stroke, and on patients with a higher median NIHSS score at admission than the general stroke population.^14–17^ Furthermore, the focus of prehospital stroke scale research has been on the identification and triage of patients with large vessel occlusion (LVO). A recent study evaluating eight prehospital LVO scales found that the National Institutes of Health Stroke Scale (NIHSS), albeit performed by emergency department clinicians, was a more accurate predictor of LVO than the prehospital scales.^18^ NIHSS captures a wider range of neurological signs and symptoms, is the stroke scale of choice in-hospital, and is the most validated stroke scale in the clinical setting.^19^ In contrast, no consensus has been reached on which scale is best suited for prehospital use.^20^

Prehospital evaluation of patients with mild minor stroke (NIHSS 0–2) is poorly studied, it is uncertain how and if prehospital stroke scales identify patients in this category. Paramedics miss up to 30% of all patients with acute stroke.^11^ This number is likely to be higher for minor stroke as the diagnostic accuracy of prehospital stroke scales increases with each extra point of NIHSS score.^21^ Finally, there is limited data on the presenting signs and symptoms of stroke mimics in a prehospital setting.

The Paramedic Norwegian Acute Stroke Prehospital Project (ParaNASPP) trial aimed to increase diagnostic accuracy and improve triage of stroke in the prehospital setting by implementing an intervention consisting of increased paramedic stroke knowledge, prehospital NIHSS, and direct, facilitated communication with stroke physicians through a mobile application (eSTROKE app).^22^ The primary endpoint of increased positive predictive value was neutral, but the stroke patients in the intervention group had lower median NIHSS at admission (NIHSS 3 vs 5), suggesting that the intervention identified patients with more subtle symptoms.

The aim of this post-hoc secondary analysis of the ParaNASPP trial was to identify and describe the prehospital presenting signs and symptoms reported by paramedics in a prehospital patient population with suspected mild minor stroke, defined as NIHSS 0–2 at hospital admission, regardless of symptoms being disabling or non-disabling. Further we aimed to reconstruct and evaluate prehospital stroke scales by using the reported prehospital signs and symptoms to calculate sensitivity, specificity, positive and negative predictive value for nine prehospital stroke scales in patients with mild minor stroke.

## Methods

### Study design

The present study is a post-hoc analysis of data from the ParaNASPP trial, a prospective, pragmatic, stepped-wedge, cluster-randomized controlled trial that took place at Oslo University Hospital (OUH), Norway from June 3, 2019 to July 1, 2021. The ParaNASPP protocol and trial results have previously been published.^22,23^ For simplicity we use the word “paramedic” for all ambulance personnel, regardless of educational level.

In the ParaNASPP trial, 267 paramedics from 5 ambulance stations, called clusters, received training through an e-learning course covering general stroke knowledge, cerebral anatomy, pathophysiology and symptoms of stroke, in-hospital treatment, and how to perform NIHSS. After completion of the online course all study participants had to complete practical NIHSS training. During this training, NIHSS and how to use the eSTROKE app were demonstrated and practiced together with experienced stroke physicians. All paramedics started in the control group, before one cluster at a time received training and crossed over to the intervention group.

In the intervention group the eSTROKE app was used to perform prehospital NIHSS and send the results directly to the on-call stroke physician, before a telephone conference was performed. The control group followed standard prehospital stroke protocol; Face, Arm, Speech, Time (FAST) test as a stroke screening tool and a telephone conference with the on-call stroke physician. During the telephone conference, the paramedics in both intervention and control groups would present the patient history, including symptoms, vital parameters, and results of the prehospital stroke scale to the stroke physician. A joint triage decision, where the stroke physician had the final say, was made based on the information provided by the paramedics.

### Patient population and presenting symptoms

Patients included in this secondary analysis had prehospital suspected mild minor stroke, defined as NIHSS 0–2 when assessed by a stroke physician at hospital admission. Patients were included regardless of disabling or non-disabling symptoms, irrespective of the final diagnosis, and whether the patients were included in the original intervention or control groups. The clinical ambulance records were collected, and prehospital signs and symptoms were registered. The records were often unclear, with vague or imprecise terms such as unwell, uncomfortable, eyesight problem, or dizzy being used, making it impossible to avoid a degree of interpretation. HFB did all the data extraction from the ambulance clinical records, when in doubt ECS was consulted. If there still was uncertainty MG and MRH were consulted. ECS, MG and MRH were blinded to NIHSS score and final diagnosis when consulted.

When dispatched to a patient with suspected stroke, paramedics registered both the subjective symptoms mentioned by the patient and the objective signs assessed by examination in the clinical ambulance records. For transient ischemic attack (TIA) patients, where the signs and symptoms had been resolved prior to the paramedic examination, the symptoms reported by the patient were recorded in the same manner.

There was great diversity in the recorded signs and symptoms. Therefore, the signs and symptoms were grouped. If a presenting sign or symptom was reported in more than 10 ambulance clinical records, this symptom was included as a single variable, whereas symptoms recorded in less than 10 patients were grouped into the variables “Other neurological signs and symptoms” (e.g. alexia, apraxia, reduced tempo, and grip strength) or “Other” (e.g. difficulty breathing, fever, and palpitations). Signs and symptoms that were deemed to be assessed as part of an NIHSS examination were categorized as NIHSS symptoms, regardless of the number of patients presenting with the symptom or examination finding. Signs and symptoms registered by other stroke scales were plotted individually, and used for the reconstruction of the scales, but otherwise reported as part of the grouped variables. See Supplemental Figure S1 for a complete list of presenting signs and symptoms, interpretation, and final variables.

### Prehospital stroke scales

**Figure 1.**
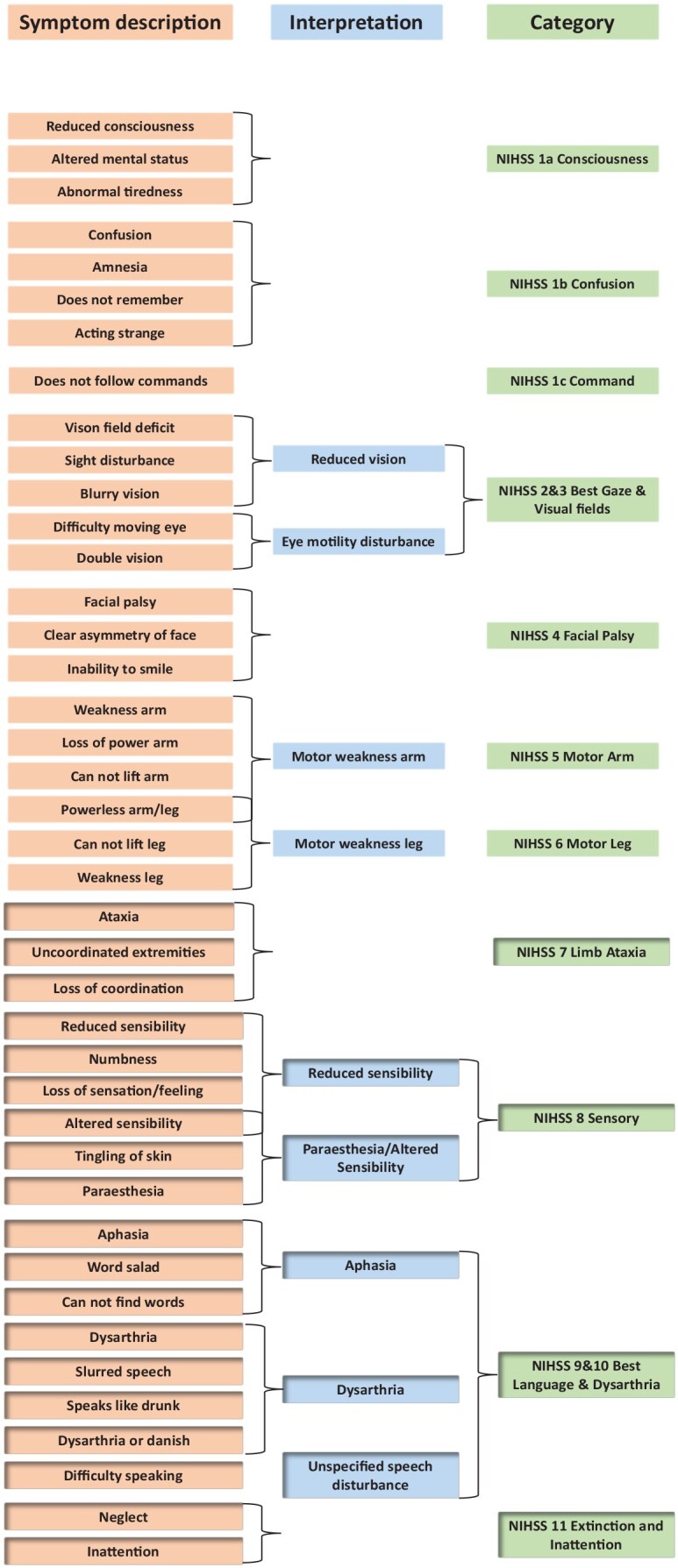
List of signs and symptoms, and how they are grouped to fit into NIHSS Items.

Scores of the prehospital stroke scales were reconstructed based on the documented prehospital presenting signs and symptoms in the clinical ambulance records. Figure 1 provides details on signs and symptoms that were classified as NIHSS symptoms.

We selected nine of the most studied prehospital stroke scales for this analysis, including full NIHSS used by paramedics in the ParaNASPP study: FAST^24^; Cincinnati Prehospital Stroke Scale (CPSS)^25^; Balance, Eyes, Face, Arm, Speech, Time (BE-FAST)^15^; Los Angeles Prehospital Stroke Screen (LAPSS)^14^; Melbourne Ambulance Stroke Screen (MASS)^26^; Medic Prehospital Assessment for Code Stroke (MedPacs)^27^; PreHospital Ambulance Stroke Test (PreHAST)^28^; and shortened National Institutes of Health Stroke Scale for Emergency Medical Services (sNIHSS-EMS).^29^  Table 1 provides an overview of the symptoms assessed by each of the selected prehospital stroke scales.

**Table 1.** Prehospital stroke scales and the symptoms evaluated by each scale.

Symptoms assessed	NIHSS^19^	FAST^24^/CPSS^25^	BE-FAST^15^	LAPSS^*14^	MASS^*26^	MedPACS^*27^	PreHAST^28^	sNIHSS-EMS^29^
Level of consciousness (LOC)	x	-	-	-	-	-	-	x
LOC question	x	-	-	-	-	-	-	-
LOC commands	x	-	-	-	-	-	x	-
Gaze	x	-	x	-	-	x	x	-
Visual	x	-	x	-	-	-	x	-
Facial palsy	x	x	x	x	x	x	x	x
Motor arm	x	x	x	x	x	x	x	x
Motor leg	x	-	x	-	-	x	x	x
Limb ataxia	x	-	-	-	-	-	-	-
Sensory	x	-	-	-	-	-	x	x
Language	x	x	x	-	x	x	x	x
Dysarthria	x	x	x	-	x	x	x	x
Extinction/inattention	x	-	-	-	-	-	x	-
Hand grip	-	-	-	x	x	-	-	-
Balance and gait	-	-	x	-	-	-	-	-

^*^Score set to not evaluated if age <45, seizure present in history, prehospital mRS ⩾4, prehospital glucose <2.8 or >22.2.

### Statistical analysis and outcome variables

The ParaNASPP trial, as a pragmatic clinical trial, used physician-based diagnosis, based both on clinical findings and advanced imaging. A final diagnosis of stroke was defined as ICD-10 (International Classification of Diseases, version 10) diagnosis code at discharge: I60 non-traumatic subarachnoid hemorrhage, I61 non-traumatic intracerebral hemorrhage, I63 cerebral infarction, I67.6 non-pyogenic thrombosis of intracranial venous system, G45.3 amaurosis fugax, G45.8 other transient cerebral ischemic attacks and related syndromes, or G45.9 transient cerebral ischemic attack, unspecified. All other discharge diagnoses were defined as stroke mimics (non-stroke).

Prehospital signs and symptoms in patients with suspected mild minor stroke (NIHSS 0–2 at hospital admission) are presented as absolute numbers and percentages of patients with a given sign or symptom. First, we compared differences in baseline characteristics in patients with NIHSS 0–2 versus NIHSS ⩾3 using χ^2^ test for categorical data and Mann-Whitney U test for comparing groups of continuous non-normally distributed data. Second, we compared presenting symptoms in patients with stroke and stroke mimics in the group with mild minor stroke using the χ^2^ test. To assess how prehospital stroke scales perform in a population with few symptoms we then evaluated the diagnostic performance of nine prehospital stroke scales, calculating sensitivity, specificity, positive predictive value (PPV), and negative predictive value (NPV) for each scale.

Finally, to evaluate if NIHSS had an unfair advantage as it was performed in the original intervention group, a sub-group analysis was done on the data from the original control group where FAST was used as a prehospital stroke scale.

### Standard protocol approvals, registrations, and patient consents

The ParaNASPP trial was registered at Clinicaltrials.gov with registration number NCT04137874. The trial was approved by the Regional Committee for Medical Research Ethics South East Norway (2018/2310). Written consent was obtained from the patient or next of kin, either during or after hospitalization.

## Results

Of the 801 patients in the ParaNASPP trial, 431 patients had suspected mild minor stroke with NIHSS 0–2 at hospital admission. Table 2 provides all baseline characteristics, while Supplemental Table S1 provides baseline characteristics grouped by NIHSS 0, 1 and 2 at admission. Supplemental Table S2 contains a comparison of baseline characteristics between stroke and stroke mimics for patients with NIHSS 0–2 at admission. Compared to patients with NIHSS ⩾3 at admission, the patients with NIHSS 0–2 were younger (68 vs 74 years old), had lower premorbid modified Rankin Scale scores and fewer stroke risk factors (atrial fibrillation, hypertension, diabetes, coronary disease, and smoking). From the original ParaNASPP intervention group 255 (57% of this material) patients had a prehospital NIHSS. There was no difference in number of signs and symptoms between the original ParaNASPP control and intervention groups. Both groups had a median of 3 (2–4) symptoms, *p* = 0.95.

**Table 2.** Baseline characteristics for patients with NIHSS 0–2 and ⩾3.

Characteristics	Total = 0–2 (*n* = 431)	NIHSS ⩾3 (*n* = 370)	*p*-value NIHSS 0–2 compared with NIHSS ⩾3
Age, years mean (SD)	68 (18)	74 (14)	<0.0001
Sex			
Women, *n* (%)	211 (49%)	169 (46%)	0.354
Men	220 (51%)	201 (54%)	0.354
Living alone	155 (36%)	139 (38%)	0.656
Currently smoking	48 (12%)	65 (19%)	0.010
Past medical history			
Atrial fibrillation	63 (15%)	87 (24%)	0.001
Hypertension	196 (45%)	197 (53%)	0.028
Hypercholesterolemia	156 (36%)	150 (41%)	0.207
Diabetes	45 (10%)	60 (16%)	0.016
Transient ischemic attack	37 (9%)	35 (9%)	0.666
Ischemic stroke	77 (18%)	83 (22%)	0.107
Coronary disease	43 (10%)	58 (16%)	0.015
Intracerebral hemorrhage	5 (1%)	15 (4%)	0.009
Anticoagulant use	76 (18%)	84 (23%)	0.074
Antiplatelet use	122 (28%)	123 (33%)	0.131
Antihypertensive use	199 (44%)	171 (46%)	0.053
Statin use	170 (40%)	143 (39%)	0.161
Premorbid modified Rankin scale 0–2	380 (88%)	260 (73%)	<0.001

Of the patients with suspected mild minor stroke, 152 (35%) had a final diagnosis of stroke or TIA, while 279 (65%) patients had a stroke mimic. Of the 152 stroke patients 81 (53%) had an ischemic stroke, 62 (41%) TIA, 7 (5%) intracerebral hemorrhage, and two (1%) subarachnoid hemorrhage.

Both stroke mimics and patients with confirmed mild minor stroke had a median (min–max) of 3 (1–8) signs and/or symptoms reported by paramedics. Symptom remission prior to the paramedics conferring the patient with the stroke physician, was reported in 66 (15.3%) patients. Of the patients with symptom remission, 35 (53%) received a final diagnosis of stroke or TIA, of which 24 (69%) were diagnosed with a TIA.

An overview of presenting signs and symptoms is shown in Table 3. Speech disturbance (aphasia, dysarthria, and unspecified speech disturbance) was the most common, present in 184 (42.7%) of the patients. While aphasia was evenly distributed among strokes and stroke mimics (23.7% vs 23.3%, *p* = 0.93), dysarthria was more prominent among the stroke patients (29.6% vs 15.8%, *p* = 0.001).

**Table 3.** Total number of patients (%) with a symptom, and number of patients (%) with a symptom given stroke or non-stroke diagnosis.

Symptoms	Total population NIHSS 0–2, *n* = 431	If stroke diagnosis *n* = 152 (35.3%)	If stroke mimic, *n* = 279 (64.7%)	*p* value for χ^2^ difference between stroke and mimic
NIHSS symptoms	380 (88.2%)	145 (95.4%)	235 (84.2%)	0.001
FAST symptoms	261 (60.6%)	114 (75.0%)	147 (52.7%)	<0.001
Consciousness	15 (3.5%)	3 (2.0%)	12 (4.3%)	0.21
Confusion	73 (16.4%)	19 (12.5%)	54 (19.4%)	0.07
Command	7 (1.6%)	3 (2.0%)	4 (1.4%)	0.7
Visual and gaze disturbance	84 (19.5%)	27 (17.8%)	57 (20.4%)	0.5
Facial palsy	104 (24.1%)	45 (29.6%)	59 (21.2%)	0.05
Motor weakness arm	69 (16.0%)	40 (26.3%)	29 (10.4%)	<0.001
Motor weakness leg	74 (17.2%)	35 (23.0%)	39 (14.0%)	0.017
Ataxia	27 (6.3%)	11 (7.24%)	16 (5.7%)	0.54
Sensory disturbance	119 (27.6%)	43 (28.3%)	76 (27.2%)	0.82
Speech disturbance^a^	184 (42.7%)	84 (55.3%)	100 (35.8%)	<0.001
Aphasia^a^	101 (23.4%)	36 (23.7%)	65 (23.3%)	0.93
Dysarthria^a^	89 (20.7%)	45 (29.6%)	44 (15.8%)	0.001
Neglect	13 (3.0%)	6 (4.0%)	7 (2.5%)	0.4
Dizziness/vertigo	160 (37.1%)	40 (26.3%)	120 (43.0%)	0.001
Headache	91 (21.1%)	22 (14.5%)	69 (24.7%)	0.013
Gait disturbance	79 (18.3%)	25 (16.5%)	54 (19.4%)	0.47
Nausea/vomiting	65 (15.1%)	10 (6.6%)	55 (19.7%)	<0.001
Syncope	17 (3.9%)	4 (2.6%)	13 (4.7%)	0.30
Other neurological^b^	47 (10.9%)	19 (12.5%)	28 (10.0%)	0.43
Other^c^	65 (15.1%)	19 (12.5%)	46 (16.5%)	0.27
Transient symptoms	66 (15.3%)	35 (23.0%)	31 (11.1%)	0.01

^a^Speech disturbance is made up of aphasia, dysarthria, and non-specific speech disturbance.

^b^Other neurological contains abnormal behavior, agraphia, alexia, apraxia/dyspraxia, ataxic gait, change/loss of taste, change/loss of hearing, difficulty swallowing, feeling of abnormal heat, leaning to one side, loss of balance, racing speech, reduced fine motor skills, reduced grip strength, reduced motor tempo, seizure, sensitivity to light, somnolence, tinnitus, and tremor.

^c^Other contains chest pain, dyspnea, nosebleed, powerless legs, fall, fever, palpitations, diarrhea, neck pain, and shoulder pain.

The second most common symptom was dizziness, present in 160 (37.1%) patients. Nearly half of the stroke mimics had dizziness as one of their symptoms (120 (43.0%)), compared with only 40 (26.3% *p* = 0.0001) of the stroke patients. Sensory disturbance and facial palsy were the third and fourth most common signs and symptoms. Sensory disturbances were equally distributed between strokes and stroke mimics, while a higher proportion of patients with stroke presented with facial palsy compared to stroke mimics (45 (29.6%) vs 59 (21.2%) *p* = 0.05).

Fifty-nine (13.7%) patients presented with only one sign or symptom. Supplemental Table S3 provides an overview of the number of patients that presented with a symptom and how often a symptom occurred together with other symptoms, both for strokes and for stroke mimics. Speech disturbance was mostly accompanied by facial palsy, arm, and leg motor weakness. Dizziness was most often the presenting complaint in patients who also had nausea and vomiting, gait disturbance and headache. Sensory disturbance was most often accompanied by symptoms not captured by any of the screened stroke scales, such as dizziness and headache. A complete list of all non-stroke diagnoses can be found in Supplemental Table S4.

Of all the stroke scales, in patients with suspected mild minor stroke, NIHSS and PreHAST had the highest sensitivity (95% (91–98) and 94% (89–97)), while NIHSS had the lowest specificity (16% (12–21)). The more complex prehospital scales such as BE-FAST and sNIHSS-EMS had high sensitivity (89% (84–94) and 88% (82–93)) and low specificity (23% (19–29) and 30% (25–36)). LAPSS had the lowest sensitivity (42% (34–50)) and highest specificity (80% (75–85)). Positive and negative predictive value varied less among the scales. Table 4 gives an overview of the performance of the different prehospital stroke scales. Sensitivity, specificity, PPV, and NPV calculated on data from the original ParaNASPP control and intervention groups can be found in Supplemental Tables S5 and S6.

**Table 4.** Reconstructed prehospital stroke scales based on presenting symptoms recorded in the ambulance clinical records (*n* = 431).

Prehospital Stroke Scale	Sensitivity (95% CI)	Specificity (95% CI)	PPV (95% CI)	NPV (95% CI)
NIHSS	95 (91–98)	16 (12–21)	38 (33–43)	86 (74–94)
FAST/CPSS	75 (67–82)	47 (41–53)	43 (38–50)	78 (71–84)
BE-FAST	89 (84–94)	23 (19–29)	39 (34–44)	80 (70–88)
LAPSS	42 (34–50)	80 (75–85)	53 (44–63)	72 (66–77)
MASS	67 (59–75)	64 (58–70)	51 (43–58)	78 (72–83)
MedPACS	73 (65–80)	47 (41–53)	43 (37–49)	76 (69–82)
PreHAST	94 (89–97)	23 (18–28)	40 (35–45)	87 (77–94)
sNIHSS-EMS	88 (82–93)	30 (25–36)	41 (35–46)	82 (74–89)

## Discussion

This post-hoc subgroup analysis of data from the ParaNASPP trial identifies and describes the presenting signs and symptoms of patients with suspected mild minor stroke, defined as NIHSS 0–2 at hospital admission, and evaluates nine prehospital stroke scales for this population.

The classic symptoms of speech disturbance, arm weakness and facial palsy were among the most common in patients with suspected mild minor strokes. This triad of symptoms was significantly more present in patients with a discharge diagnosis of stroke, whereas the patients with a stroke mimic diagnosis more often had non-focal symptoms like dizziness, headache, and nausea/vomiting. The distribution of symptoms in our study is generally in line with previous populations-based studies.^30,31^ We do, however, note that our population has a higher proportion of stroke patients with speech disturbance (55.3% vs 37.9%–39%) dizziness (26.3%, compared to 12.3%), and sensory symptoms (28.3% vs 18.9%). We also have less motor weakness arm (26.3% vs 39.1%–49%). In our trial there are fewer patients presenting with only one sign or symptom, 13.7% vs 26% in the Rotterdam study.^30^ Since the Rotterdam study was a retrospective data collection, this might be influenced by a recall bias in the patients included, whereas our data collection was from the prehospital paramedic records. In the Norwegian healthcare system paramedics have a right to admit patients, but a phone conference with the admitting physician is used to find the most suitable destination for a patient, providing the stroke physicians with an option to refuse patients they believe do not have a stroke. This might lead paramedics to embellish symptoms to increase the chance of getting a patient they believe has a stroke accepted for stroke evaluation, leading to an inclusion bias.

Only 7 (4.6%) patients with stroke in this study had symptoms not assessed by NIHSS, giving NIHSS a sensitivity of 95.4%. This is the highest among the evaluated prehospital stroke scales and probably of all prehospital stroke scales. The high sensitivity comes at a cost of low specificity and low PPV. The fact that the vast majority (84.2%) of stroke mimics also have symptoms evaluated by NIHSS, illustrates the difficulties of differentiating stroke and stroke mimics.

This considerable overlap in signs and symptoms between stroke patients and mimics must be acknowledged. The challenge is to achieve accurate field identification of those patients in need of acute stroke assessment, which is crucial for prognosis. Misdiagnosis of stroke is more frequent in patients with atypical or mild strokes.^32^ A considerable proportion of the United States population has undiagnosed stroke: The prevalence of stroke symptoms, and possibly a missed stroke diagnosis, in persons without a stroke diagnosis has been estimated to 18%,^33^ while most studies evaluating silent brain infarctions using magnetic resonance imaging find a prevalence of 10%–20%,^34^ and an estimated 14% of strokes being misdiagnosed in emergency departments.^35^ The combination of minor stroke being the most common and easiest to misdiagnose indicates that many patients miss out on optimal acute treatment, secondary prevention and rehabilitation which in turn will affect the patient prognosis.

Patients with mild minor stroke present with a wide and diverse range of symptoms in the prehospital field, and not just the classic symptoms captured by the prehospital stroke scales. Stroke patients with NIHSS 0–2 at admission are poorly described in the prehospital setting, and the prehospital stroke scales have rarely been validated for patients with minor strokes. Although these strokes are difficult to accurately triage, correct prehospital identification is often the most plausible path to acute therapy. Currently the treatment decision hinge on the vague premise of symptoms being disabling or non-disabling.^9^ This treatment should not be left to chance, and the responsibility of differentiating between non-disabling and disabling minor strokes should not be placed upon paramedics.

Though the neurological deficits in minor stroke are less disabling than those of moderate or large strokes, treatment and secondary prevention are still warranted. There is a significant risk of deterioration and recurrent events,^36,37^ and a significant 1- and 5-year risk of cardiovascular events and death.^38,39^ Furthermore, as many as two-thirds of the minor stroke patients suffer hidden impairments.^40^ Up to 40% of minor stroke patients do not return to full time paid work,^41^ up to 30% need assistance with ambulation at discharge, and an equal percentage are not discharged home.^4^ For a group of patients that is younger, has fewer comorbidities and lower premorbid mRS scores, the consequences of minor stroke are absolutely not minor, as it causes morbidity and mortality and comes with a considerable risk of stroke recurrence. It might be devastating for the individual and their families, and also costly for society in general.

The major strength of this study is that the data is prospectively collected directly from a real-world suspected stroke population, and directly from the paramedics assessing the patients. When it comes to prehospital identification of stroke patients, one can generally surmise that the more complex prehospital stroke scales, such as NIHSS, PreHAST, BE-FAST, and sNIHSS-EMS, assess more signs and symptoms, and therefore have the potential to identify more stroke patients. The shorter scales, such as FAST, CPSS, and LAPSS identify fewer patients, but a higher proportion of the patients identified will have been correctly identified as having a stroke. While the more complex scales might lead to increased strain on resources in hospitals, the simpler scales might lead to patients missing important treatment and secondary prevention, after having been in touch with the healthcare system. The fact that the original ParaNASPP control and intervention groups have the same number of signs and symptoms, when one group uses FAST and the other NIHSS, suggests that paramedics pick up on many symptoms, but not through a structured, objective examination. It is also interesting to note the high sensitivity of NIHSS in the original ParaNASPP control group (98% (90–100)), while the specificity is still low (14% (8–21)), indicating that paramedics using FAST still assess patients for symptoms included in NIHSS, but without training or competence in interpreting these symptoms.

Studies assessing other prehospital stroke scales have lacked a focus on minor stroke, and with fewer symptoms in our population we would expect a lower sensitivity. The specificity would likely be higher due to a reduced likelihood of falsely identifying a stroke for the various scales with decreasing symptom burden. It is therefore a bit surprising that we find a similar sensitivity and specificity compared to previous studies for FAST/CPSS and BE-FAST,^26,27,42–44^ a similar sensitivity and lower specificity for PreHAST and sNIHSS-EMS,^28,29,45^ and that LAPSS and MASS both had lower sensitivity and specificity.^14,26,42,46^ MedPACS had a similar sensitivity, but as we expected for all the scales, a higher specificity.^27^ Along with the low burden of symptoms in our population, the high proportion of stroke mimics might also have influenced these findings.

Compromising between prehospital high sensitivity and thereby increased strain, and high specificity with its missed treatment opportunities is not easy, but future technologies such as point-of care biomarkers or artificial intelligence might help. More research on prehospital stroke identification and triage is needed, and not only for the mild minor strokes. For now, in resource-rich healthcare systems, with well-educated and trained paramedics, the job of the prehospital services should be to provide as high a sensitivity as possible, i.e., finding all patients with suspected stroke. Through direct communication with the stroke physicians, the specificity could be increased by ruling out those without a stroke, ideally before they reach hospital. NIHSS can bridge and facilitate this communication as it provides a common language between the pre- and in-hospital parts of the acute stroke chain.^47^

Due to the overlapping nature of signs and symptoms between stroke and stroke mimics, relying solely on a dogmatic stroke scale might not be the best option. Educating and training paramedics not only to assess symptoms of stroke, but to include risk factors, pathophysiology, acute characteristics of both stroke and stroke mimics in their assessment could improve specificity and diagnostic accuracy.^48^ For LVO recognition, the clinical judgment of stroke physicians has shown to be at least equal to prehospital stroke scales.^49^ To fully utilize the clinical judgment of stroke physicians, the information from paramedics needs to be of high quality, and this may be improved through education and training. And just like every stroke patient deserves optimal treatment, are our paramedics also entitled to optimal tools for stroke recognition.

### Limitations

All patients included in this study had a continued suspected stroke diagnosis after consultation with a specialized in-hospital stroke team and represent a selected subgroup. The selection of our population is based on NIHSS at hospital admission and might be a slightly different population than the patients with prehospital NIHSS 0–2. This was done to maximize the number of patients included, as the original ParaNASPP control group did not have a prehospital NIHSS.

False negative and true negative stroke cases from the prehospital setting are not included in the analyses, as they were never included in the trial, and this may have influenced the performance of the stroke scales. Neither has any of the stroke scales evaluated been tested in-vivo on all included patients. Some items of NIHSS have high interrater variability and this may have contributed to the low specificity of the scale. To reduce the interrater variability some scales, for example, PreHAST, uses a simplified scoring system compared to NIHSS, and might therefore have benefited from real world testing.

The use of NIHSS and FAST in the ParaNASPP trial could influence how the paramedics assessed and reported symptoms. Grip strength may have been a more prominent symptom but might have been reported as motor weakness arm and not specifically as grip strength. This may have led to an underestimation of the performance of LAPSS and MASS. The performance of NIHSS might have been affected by symptoms, such as double vision and the diffuse “eyesight problem,” that do not necessarily give point as part of NIHSS, but are here included as they probably would have been detected during assessment of gaze and visual fields. Some signs and symptoms might have been overlooked by paramedics and therefore not recorded in the ambulance clinical records.

A final diagnosis of stroke was made following clinical practice at OUH, and MRI was used in the diagnosis at the discretion of the stroke physician responsible for the patient during hospitalization. This might have led to a tendency of diagnosing TIA or minor stroke in those with more classical symptoms, leading to a type of confirmation bias that would favor the shorter stroke scales that focus more on the classical symptoms.

## Conclusion

For patients included in the ParaNASPP trial with suspected mild minor stroke, defined as NIHSS 0–2 at hospital admission, there was significant overlap in prehospital signs and symptoms between stroke and stroke mimics, indicating the difficulty of correct triage. Only 35% of the patients with suspected mild minor stroke had a final diagnosis of stroke or TIA. Prehospital stroke scales had either high sensitivity or high specificity indicating that relying solely on clinical scales to identify patients with real minor strokes is insufficient. NIHSS and PreHAST had the highest sensitivity of all the screened prehospital stroke scales, indicating that most minor strokes would be identified, albeit at a cost of a high rate of false positives. Yet none of the scales tested are sufficient on their own to identify and triage suspected stroke patients, therefore knowledge and competence about when to use and how to interpret a prehospital stroke scale, together with increased general stroke knowledge, is paramount and might help reduce the overtriage. A prehospital stroke scale that enables field identification of minor strokes, provides a common language between paramedics and stroke physicians, and facilitates correct triage of these patients is crucial for an optimal stroke service.

## Supplementary Material

sj-pdf-1-eso_23969873251360592

## Data Availability

The data is available for the researchers in this study and can be made available for new projects upon reasonable request and provided ethical approval is granted.

## Non-standard abbreviations and acronyms

ParaNASPP – Paramedic Norwegian Acute Stroke Prehospital Project

DAPT – Dual Antiplatelet Treatment

NIHSS – National Institutes of Health Stroke Scale

OUH – Oslo University Hospital

FAST – Face Arm Speech Time

CPSS – Cincinnati Prehospital Stroke Scale

BE-FAST – Balance Eyes Face Arm Speech Time

LAPSS – Los Angeles Prehospital Stroke Screen

MASS – Melbourne Ambulance Stroke Screen

MedPACS – Medic Prehospital Assessment for Code Stroke

PreHAST – PreHospital Ambulance Stroke Test

sNIHSS-EMS – shortened National Institutes of Health Stroke Scale for Emergency Medical Services

## Trial registration

Clinicaltrials.gov: NCT04137874.
