# Supplementary material for: Presenting symptoms and diagnostic accuracy of prehospital stroke scales for patients with suspected mild minor stroke
Source: Eur Stroke J. 2026 Jan 1;11(1):23969873251360592. doi: 10.1093/esj/23969873251360592 (PMC12866222; doi:10.1093/esj/23969873251360592)
Supplement: sj-pdf-1-eso_23969873251360592 [file sj-pdf-1-eso_23969873251360592.pdf]

Supplementary material

Table S1 *Baseline characteristics of patients with suspected minor stroke grouped by NIHSS as admission.*

**Characteristics**	**NIHSS = 0**		**NIHSS = 1**		**NIHSS = 2**	
	n = 224		n = 109		n = 98	
Age, years mean (SD)	67	(18)	68	(17)	71	(17)
Sex						
Women, n (%)	112	(50%)	50	(46%)	49	(50%)
Men	112	(50%)	59	(54%)	49	(50%)
Past medical history						
Atrial fibrillation	23	(10%)	20	(18%)	20	(20%)
Hypertension	92	(41%)	48	(44%)	56	(57%)
Hypercholesterolemia	75	(33%)	40	(37%)	41	(42%)
Diabetes	18	(8%)	11	(10%)	16	(16%)
Transitory Ischemic Attack	19	(8%)	9	(8%)	9	(9%)
Ischemic stroke	30	(13%)	22	(20%)	25	(26%)
Coronary disease	19	(8%)	13	(12%)	11	(11%)
Intracerebral hemorrhage	2	(1%)	2	(2%)	1	(1%)
Anticoagulant use	31	(13%)	26	(24%)	19	(19%)
Antiplatelet use	58	(26%)	31	(28%)	33	(34%)
Antihypertensive use	142	(40%)	75	(33%)	75	(33%)
Statin use	80	(36%)	44	(40%)	46	(47%)
Currently smoking	21	(10%)	16	(16%)	11	(12%)
Living alone	75	(33%)	39	(36%)	41	(42%)
Premorbid modified Rankin Scale 0-2	209	(95%)	95	(90%)	76	(81%)

Table S2 Comparison of b*aseline characteristics between stroke patients and stroke mimics for with NIHSS 0-2 at admission.*

**Characteristics**	**Stroke** n = 152		**Non-stroke** n = 279		**p-value**
Age, years mean (SD)	75	(12)	64	(19)	0.30
Sex					
Women, n (%)	68	(45%)	143	(51%)	0.20
Men	84	(55%)	136	(49%)	0.20
Past medical history					
Atrial fibrillation	32	(21%)	31	(11%)	0.005
Hypertension	86	(57%)	110	(39%)	0.001
Hypercholesterolemia	62	(41%)	94	(34%)	0.14
Diabetes	22	(14%)	23	(8%)	0.043
Transitory Ischemic Attack	15	(10%)	22	(8%)	0.48
Ischemic stroke	30	(20%)	47	(17%)	0.45
Coronary disease	14	(9%)	29	(10%)	0.69
Intracerebral hemorrhage	1	(1%)	4	(1%)	0.47
Anticoagulant use	32	(21%)	44	(16%)	0.17
Antiplatelet use	44	(29%)	78	(28%)	0.83
Antihypertensive use	77	(51%)	93	(33%)	<0.001
Statin use	58	(38%)	88	(32%)	0.16
Currently smoking	20	(14%)	28	(11%)	0.43
Living alone	60	(40%)	95	(34%)	0.24
Premorbid modified Rankin Scale 0-2	136	(91%)	244	(90%)	0.68

Table S3 *Number of patients with a combination of symptoms, for both stroke patients and non-stroke patients.*

	NIHSS symptoms	Fast symptoms	Consciousness	Confusion	Visual and gaze disturbance	Facial palsy	Motor weakness arm	Motor weakness leg	Ataxia	Sensory disturbances	Speech disturbance	Aphasia	Dysarthria	Neglect	Dizziness/Vertigo	Headache	Gait disturbance	Nausea/Vomiting	Syncope	Other neurological	Other	Suspected TIA
NIHSS symptoms	145/235	114/261	3/15	19/73	27/84	45/104	40/69	35/74	11/27	43/119	85/186	36/101	45/89	6/13	35/115	21/83	21/55	7/35	4/16	18/44	4/59	35/66
Fast symptoms	147/261	114/147	3/11	15/47	16/44	45/104	40/69	29/56	10/18	26/66	85/186	36/101	45/89	2/7	18/55	13/46	16/39	2/9	3/12	13/27	3/14	31/54
Consciousness	12/15	8/11	3/12	1/5	0/2	1/4	2/4	2/4	0/2	0/1	3/9	1/6	1/4	0/1	0/5	0/1	1/1	0/1	1/3	0/4	1/1	1/1
Confusion	54/73	32/47	4/5	19/54	5/11	4/13	4/7	5/7	1/3	0/6	11/35	5/26	5/12	3/3	3/11	2/11	1/9	0/6	1/2	5/10	2/9	4/10
Visual and gaze	57/84	28/44	2/2	6/11	27/57	6/11	4/9	7/12	2/5	5/16	11/34	5/23	5/11	1/2	11/35	4/29	3/9	2/13	1/2	5/10	1/3	5/17
Facial palsy	59/104	59/104	3/4	9/13	5/11	45/59	14/20	10/19	4/9	7/29	33/60	9/20	21/37	1/5	8/19	4/17	11/18	0/4	1/7	6/12	1/4	15/21
Motor weakness arm	29/69	29/69	2/4	3/7	5/9	6/20	40/29	20/40	7/9	12/23	17/29	4/11	12/18	1/3	7/20	8/17	5/8	0/1	1/3	6/9	1/4	7/9
Motor weakness leg	39/74	27/56	2/4	2/7	5/12	9/19	20/40	35/39	5/9	7/19	17/32	5/10	10/20	1/3	9/24	6/17	4/8	2/7	2/4	3/7	1/6	8/8
Ataxia	16/27	8/18	2/2	2/3	3/5	5/9	2/9	4/9	11/16	4/9	5/12	2/6	2/5	2/3	3/8	3/8	1/3	0/1	0/0	1/4	0/1	0/0
Sensory disturbances	76/119	40/66	1/1	6/6	11/16	22/29	11/23	12/19	5/9	43/76	16/36	3/18	11/20	1/3	19/50	11/41	7/14	4/11	0/5	4/15	0/4	8/14
Speech disturbance	101/186	101/186	6/9	24/35	23/34	27/60	12/29	15/32	7/12	20/36	85/101	36/101	45/89	1/3	9/31	8/32	12/30	2/6	3/5	10/20	3/9	25/44
Aphasia	65/101	65/101	5/6	21/26	18/23	11/20	7/11	5/10	4/6	15/18	65/101	36/65	6/19	1/3	3/15	2/18	3/12	1/3	2/4	4/10	2/4	10/23
Dysarthria	44/89	44/89	3/4	7/12	6/11	16/37	6/18	10/20	3/5	9/20	44/89	13/19	45/44	0/0	6/16	6/16	9/18	1/3	1/2	5/10	1/5	14/17
Neglect	7/13	5/7	1/1	0/3	1/2	4/5	2/3	2/3	1/3	2/3	2/3	2/3	0/0	6/7	3/7	1/1	0/1	1/1	0/1	1/3	1/1	0/2
Dizziness/Vertigo	80/115	37/55	5/5	8/11	24/35	11/19	13/20	15/24	5/8	31/50	22/31	12/15	10/16	4/7	40/120	10/44	11/47	10/59	2/8	3/12	2/10	3/12
Headache	62/83	33/46	1/1	9/11	25/29	13/17	9/17	11/17	5/8	30/41	24/32	16/18	10/16	0/1	34/44	22/69	5/14	2/15	0/1	2/9	1/5	0/7
Gait disturbance	34/55	23/39	0/1	8/9	6/9	7/18	3/8	4/8	2/3	7/14	18/30	9/12	9/18	1/1	36/47	9/14	25/54	3/24	0/1	2/3	1/3	1/3
Nausea/Vomiting	28/35	7/9	1/1	6/6	11/13	4/4	1/1	5/7	1/1	7/11	4/6	2/3	2/3	0/1	49/59	13/15	21/24	10/55	1/3	1/4	1/5	0/1
Syncope	12/16	9/12	2/3	1/2	1/2	6/7	2/3	2/4	0/0	5/5	2/5	2/4	1/2	1/1	6/8	1/1	1/1	2/3	4/13	0/1	0/1	0/2
Other neurological	26/44	14/27	4/4	5/10	5/10	6/12	3/9	4/7	3/4	11/15	10/20	6/10	5/10	2/3	9/12	7/9	1/3	3/4	1/1	19/28	0/1	2/3
Other	18/59	11/14	0/1	7/9	2/3	3/4	3/4	5/6	1/1	4/4	6/9	2/4	4/5	0/1	8/10	4/5	2/3	4/5	1/1	1/1	5/21	0/0
Suspected TIA	31/66	23/54	0/1	6/10	12/17	6/21	2/9	0/8	0/0	6/14	19/44	13/23	3/17	2/2	9/12	7/7	2/3	1/1	2/2	1/3	0/0	35/31

Green writing is number of patients with a stroke diagnosis with a combination of symptoms given as proportion of total patients with this symptom combination. Red writing is number of patients with a non-stroke diagnosis with a combination of symptoms given as proportion of total patients with this symptom combination. In panel where a symptom "meets itself" the total number of stroke and non-stroke patients with a given symptom is provided (green for stroke, red for non-stroke).

Table S4 *List of all non-stroke ICD-10 diagnoses at hospital discharge*

ICD 10 Code	Number of patients with diagnosis
C71.4 Malignant neoplasm of occipital lobe	1
C79.3 Secondary malignant neoplasm of brain and cerebral meninges	1
D 59.1 Anemia	1
E86 Volume depletion	3
E87.6 Hypokalemia	1
F05 Delirium due to known physiological condition	1
F06 Mild neurocognitive disorder due to known physiological condition	1
F10 Alcohol related disorders	5
F15 Opioid related disorders	1
F23 Brief psychotic disorder	1
F41 Anxiety disorder	2
F45 Somatoform disorder	1
G20 Parkinson's disease	5
G30 Alzheimer's disease	1
G40 Epilepsy and recurrent seizures	10
G43 Migraine with aura	30
G45.1 Carotid artery syndrome (hemispheric)	1
G45.4 Transient global amnesia	11
G51 Bell's palsy	8
G55 Nerve root and plexus compressions	1
G56 Mononeuropathies of upper limb	1
G65 Polyneuropathies and other disorders of the peripheral nervous system	1
G83 Monoplegia of upper limb	3
G96 Disorder of central nervous system, unspecified	1
H81 Disorders of vestibular function	1
H46 Optic neuritis	1
H53 Subjective visual disturbances	5
H66 Acute suppurative otitis media	1
H81.0 Ménière's disease	1
H81.1 Benign paroxysmal vertigo	3
H81.2 Vestibular neuronitis	10
H81.3 Other peripheral vertigo	2
H81.8 Other disorders of vestibular function	3
I10 Essential (primary) hypertension	3
I21 Acute myocardial infarction	1
I26 Pulmonary embolism	1
I48 Paroxysmal atrial fibrillation	2
I49 Sick sinus syndrome	1
I66.2 Occlusion and stenosis of posterior cerebral artery	1
I67.4 Hypertensive encephalopathy	1
I67.5 Moyamoya	1
I69 Sequelae of cerebrovascular disease	6
I72.6 Thoracoabdominal aortic aneurysm, without rupture	1
I95.1 Hypotension	5
J02 Acute pharyngitis	1
J12 Viral pneumonia	1
J15 Bacterial pneumonia	1
K20 Esophagitis	1
K26 Duodenal ulcer	1
K74.6 Other and unspecified cirrhosis of liver	1
M15 Polyosteoarthritis	1
M54 Radiculopathy	1
M79 Myalgia	3
N10 Acute pyelonephritis	1
N39 Urinary tract infection	4
Q67 Congenital facial asymmetry	1

R00.1 Tachycardia, unspecified	1
R00.1 Bradycardia, unspecified	1
R06.4 Hyperventilation	1
R20.1 Hypoesthesia of skin	1
R20.2 Paresthesia of skin	9
R27 Ataxia, unspecified	1
R29.8 Other symptoms and signs involving the nervous and musculoskeletal systems	18*
R33 Retention of urine	1
R41 Disorientation, unspecified	3
R42 Dizziness and giddiness	43
R47.0 Dysphasia and aphasia	4
R47.1 Dysarthria and anarthria	8
R47.8 Other speech disturbances	2
R51 Headache	17
R55 Syncope and collapse	3
S06.0 Concussion	1
S06.5 Traumatic subdural hemorrhage	1
S09 Unspecified injury of face and head	1
Y48 Side effect of local anesthetics	1
Z03.3 Observation for suspected nervous system disorder	7

** Includes 7 patients where the ICD-10 diagnosis Z03.3 Observation for suspected nervous system disorder was wrongly used.*

Table S5 *Reconstructed prehospital stroke scales based on presenting symptoms recorded in the ambulance clinical records from data collected from the original ParaNASPP control group. n=176*

	**Sensitivity**	**Specificity**	**PPV**	**NPV**
NIHSS	98 (90-100)	14 (8-21)	34 (26-42)	94 (73-100)
FAST/CPSS	76 (62-87)	47 (38-56)	39 (29-49)	81 (70-90)
BE-FAST	91 (80-97)	23 (16-31)	34 (27-43)	85 (68-95)
LAPSS	37 (24-51)	82 (74-88)	48 (32-64)	75 (66-82)
MASS	65 (51-77)	66 (56-74)	45 (34-57)	81 (72-88)
MedPACS	70 (56-82)	52 (43-62)	40 (30-50)	80 (70-88)
PreHAST	96 (87-100)	20 (13-30)	35 (27-43)	92 (75-99)
sNIHSS-EMS	94 (85-99)	30 (22-38)	37 (29-46)	92 (80-98)

Table S6 *Reconstructed prehospital stroke scales based on presenting symptoms recorded in the ambulance clinical records from data collected from the original ParaNASPP intervention group. n=255*

	**Sensitivity**	**Specificity**	**PPV**	**NPV**
NIHSS	94 (87-98)	17 (12-24)	41 (35-48)	82 (65-93)
FAST/CPSS	75 (65-83)	48 (40-56)	47 (39-55)	75 (65-83)
BE-FAST	89 (81-94)	24 (17-31)	42 (35-49)	77 (63-88)
LAPSS	45 (35-55)	78 (71-85)	56 (45-68)	70 (62-76)
MASS	68 (58-77)	63 (55-71)	54 (45-53)	76 (68-83)
MedPACS	75 (65-83)	43 (35-51)	45 (37-53)	73 (63-82)
PreHAST	93 (86-97)	25 (18-32)	44 (37-51)	85 (71-94)
sNIHSS-EMS	85 (76-91)	31 (24-38)	43 (36-51)	76 (64-86)

Figure S1 *List of all presenting symptoms, how they were interpreted and combined into final categories.*

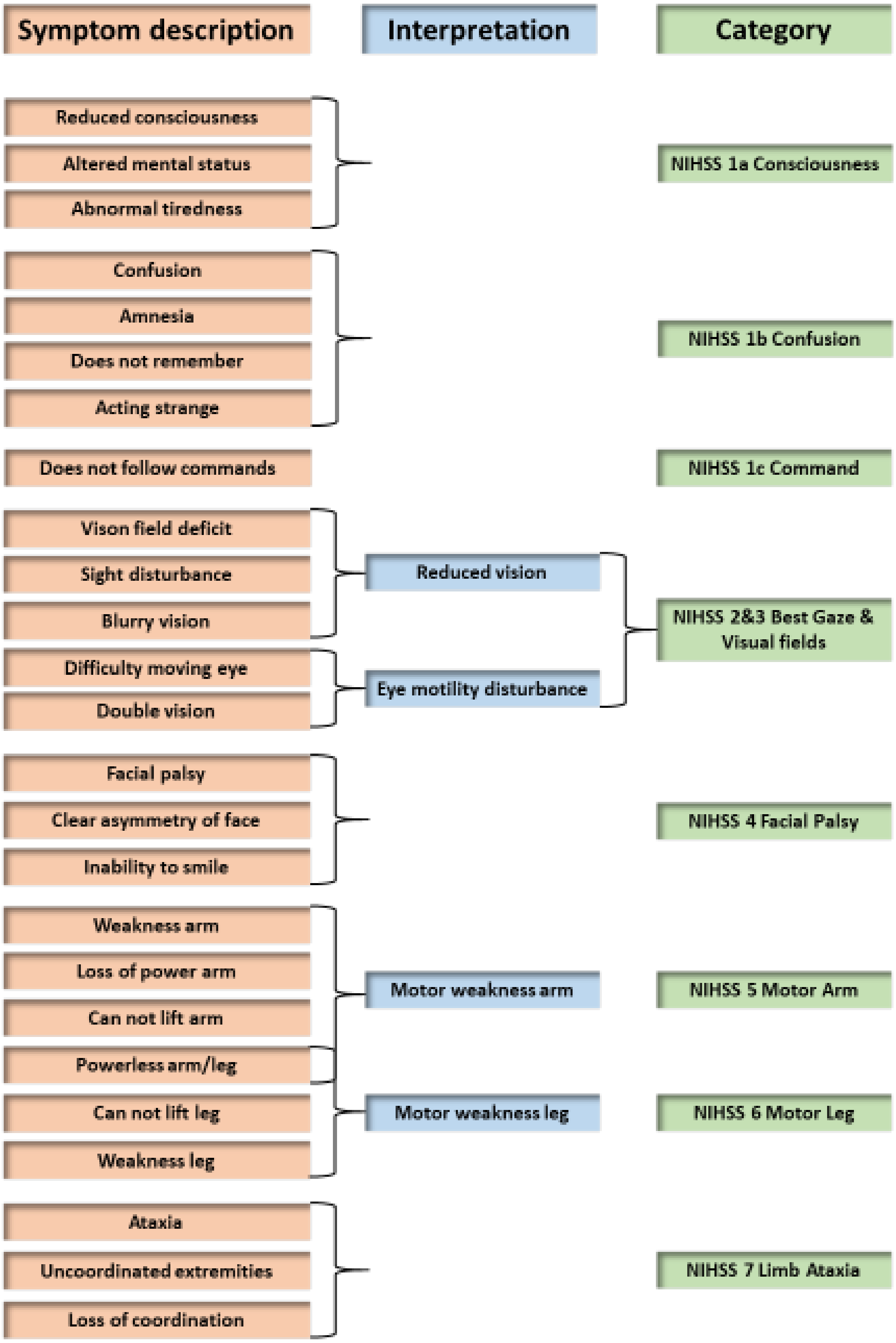

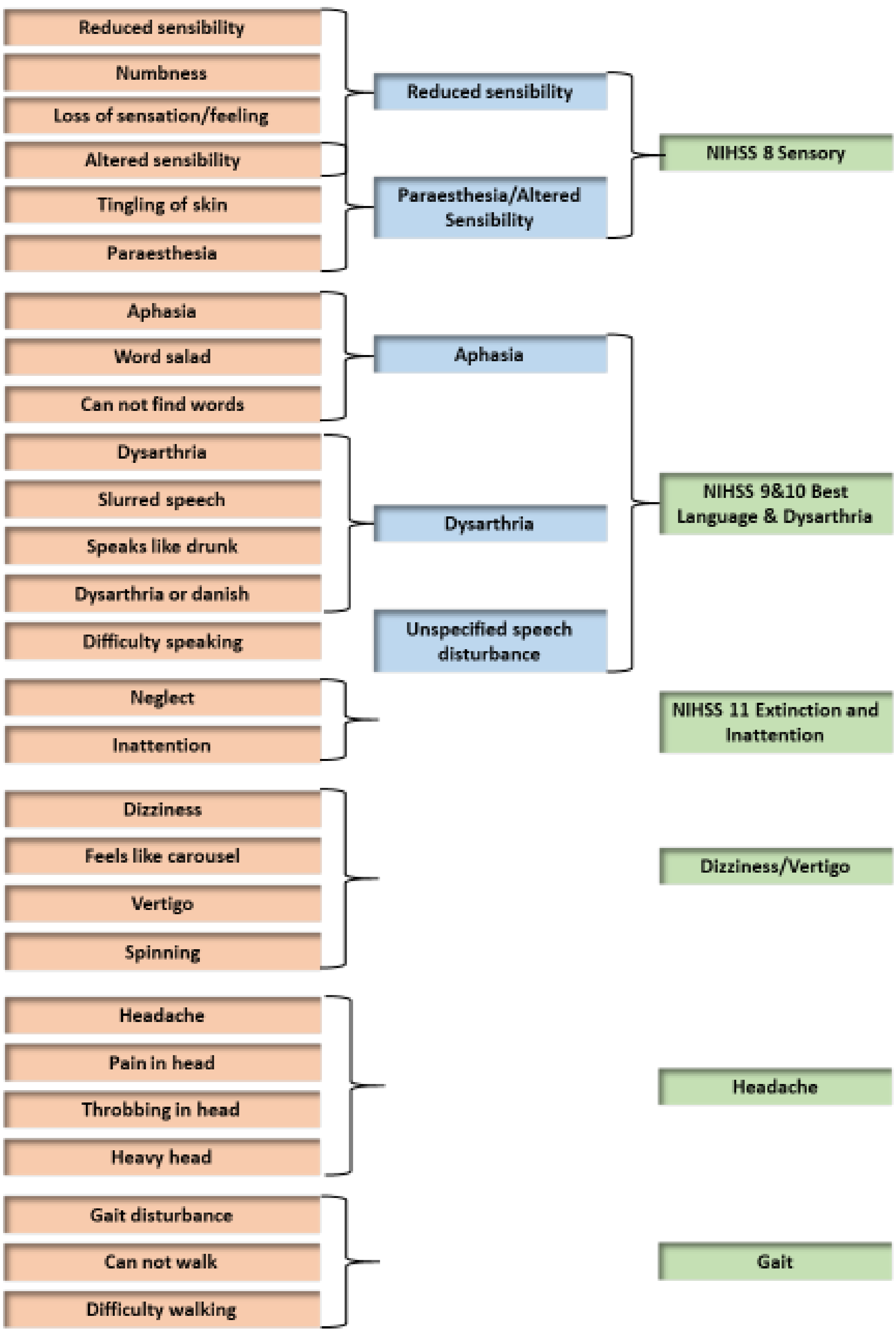
Reduced sensibility
Numbness
Loss of sensation/feeling
Altered sensibility
Tingling of skin
Paraesthesia
Reduced sensibility
Paraesthesia/Altered Sensibility
NIHSS 8 Sensory
Aphasia
Word salad
Can not find words
Dysarthria
Slurred speech
Speaks like drunk
Dysarthria or danish
Difficulty speaking
Aphasia
Dysarthria
Unspecified speech disturbance
NIHSS 9&10 Best Language & Dysarthria
Neglect
Inattention
NIHSS 11 Extinction and Inattention
Dizziness
Feels like carousel
Vertigo
Spinning
Dizziness/Vertigo
Headache
Pain in head
Throbbing in head
Heavy head
Headache
Gait disturbance
Can not walk
Difficulty walking
Gait

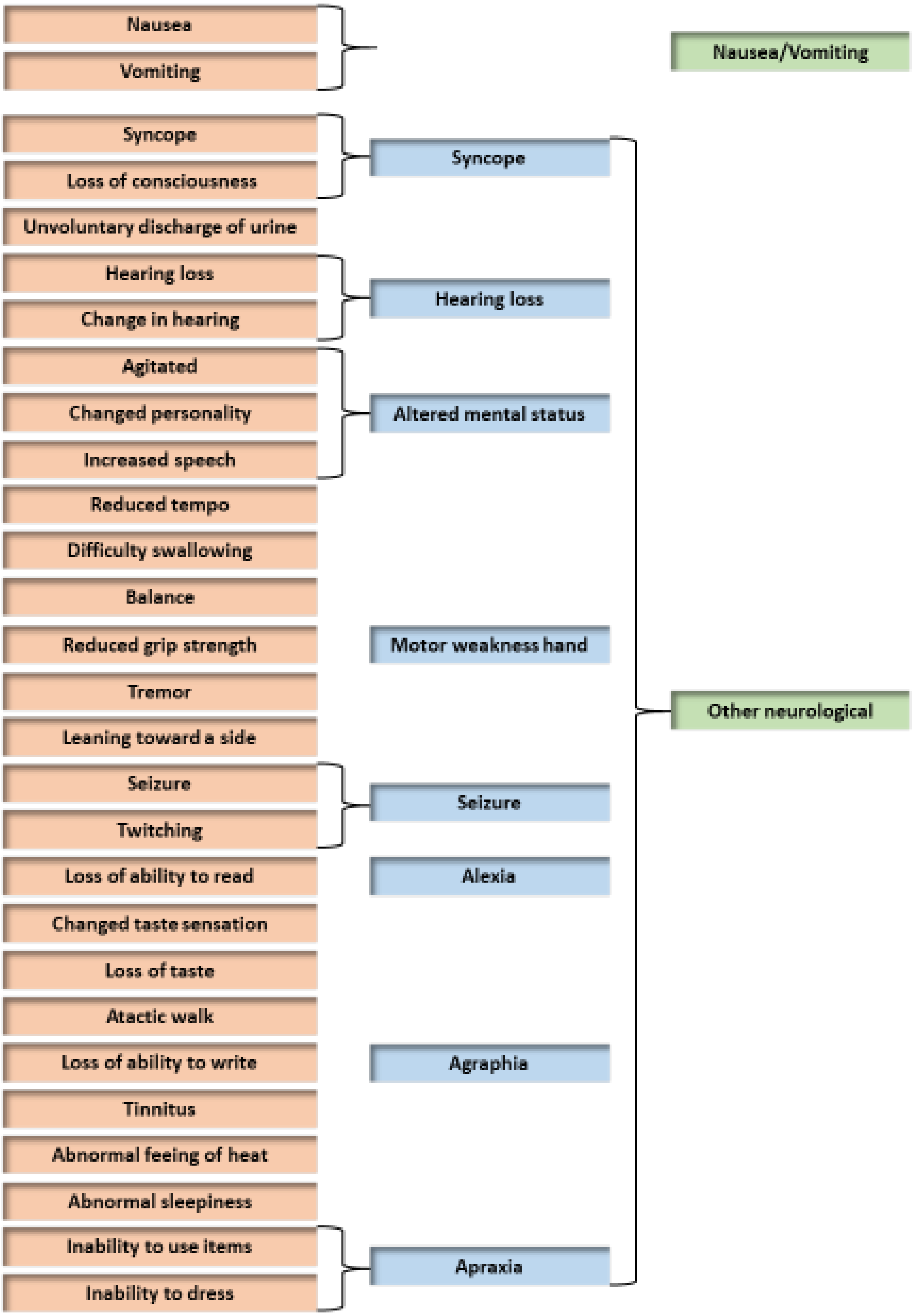
Nausea
Vomiting
Syncope
Loss of consciousness
Unvoluntary discharge of urine
Hearing loss
Change in hearing
Agitated
Changed personality
Increased speech
Reduced tempo
Difficulty swallowing
Balance
Reduced grip strength
Tremor
Leaning toward a side
Seizure
Twitching
Loss of ability to read
Changed taste sensation
Loss of taste
Atactic walk
Loss of ability to write
Tinnitus
Abnormal feeing of heat
Abnormal sleepiness
Inability to use items
Inability to dress
Syncope
Hearing loss
Altered mental status
Motor weakness hand
Seizure
Alexia
Agraphia
Apraxia
Nausea/Vomiting
Other neurological

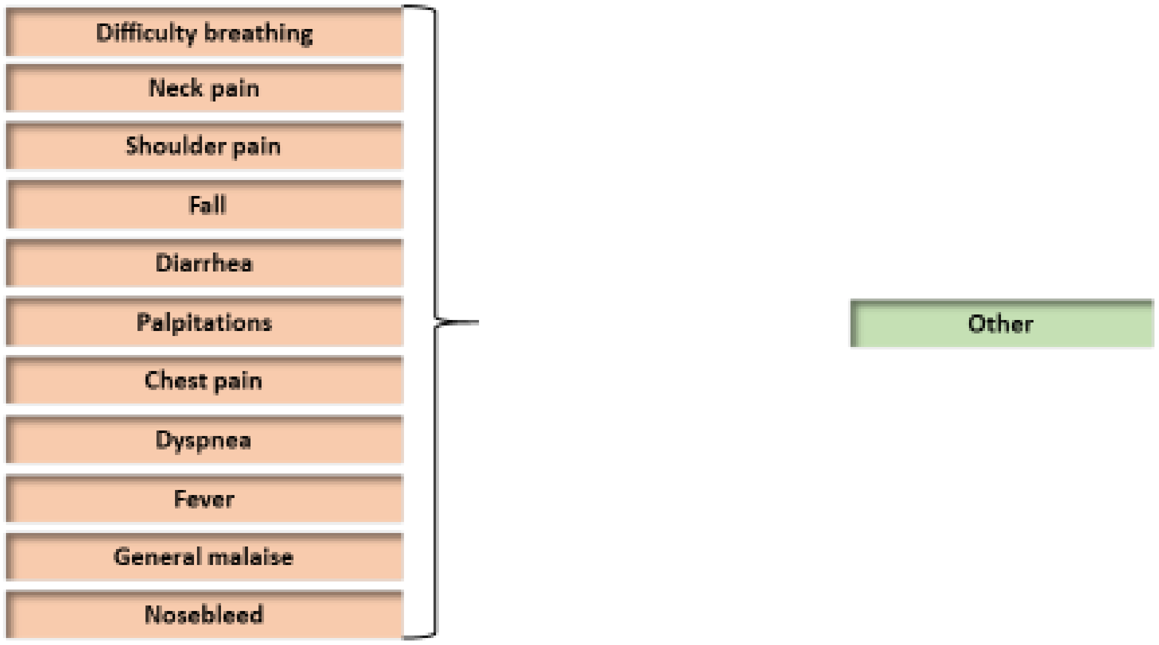

As it is difficult to differentiate between dizziness and vertigo, these were combined to one variable. Similarly, visual disturbances were grouped together including disturbance of gaze, double vision, visual field deficits, and the unspecified “vision disturbance”. Sensory disturbances include both reduced sensibility and paresthesia as vague the ambulance clinical records often used terms that made it impossible to differentiate between the two were. Many patients with motor weakness of the lower limbs would most likely have difficulties walking, but to limit interpretation of symptoms, gait difficulty was only plotted when explicitly described by paramedics. Suspected TIA was registered when the paramedics wrote this as a tentative diagnosis or explicitly described the symptoms as no longer present.
